# Convenient, high-efficiency multiplex genome editing in autotetraploid alfalfa using endogenous *U6* promoters and visual reporters

**DOI:** 10.1007/s42994-025-00200-z

**Published:** 2025-02-10

**Authors:** Xiuzhi Xia, Shihao Li, Na Wang, Panxu Cheng, Butuo Zhu, Pengcheng Zhang, Dahai Yang, Hao Lin, Lifang Niu

**Affiliations:** 1 Biotechnology Research Institute, Chinese Academy of Agricultural Sciences, Beijing, 100081 China; 2 College of Grassland Science, Shanxi Agricultural University, Jinzhong, 030801 China; 3 Tobacco Breeding and Biotechnology Research Center, Yunnan Academy of Tobacco Agricultural Sciences, Key Laboratory of Tobacco Biotechnological Breeding, National Tobacco Genetic Engineering Research Center, Kunming, 650021 China

**Keywords:** Alfalfa, Hairy root system, *MsU6* promoter, Multiplex genome editing, Visual reporters

## Abstract

CRISPR/Cas is a simple, robust, versatile tool for plant biology studies and precision plant breeding. However, establishing a high-efficiency gene editing system for multiplex editing of the autotetraploid crop alfalfa (*Medicago sativa* L.), the most important forage legume worldwide, remains a formidable challenge. Here, we systematically identified endogenous *U6* promoters in alfalfa through transient expression via *Agrobacterium*-mediated infiltration of alfalfa leaves. We further demonstrated the efficacy of the three most active promoters for genome editing using an optimized alfalfa hairy root system. Subsequently, we established an improved CRISPR/Cas9 multiplex system containing three or four tandemly arrayed *MsU6*-promoter-driven polycistronic tRNA-sgRNA (PTG) expression cassettes, each consisting of three tRNA-sgRNA units, to simultaneously edit three or four alfalfa genes, coupled with the visual reporter *RH1* or *RUBY*. This toolkit showed efficient multiplex editing in the hairy root system with visual selection. We successfully obtained regenerated, red-colored shoots resulting from the stable transformation of alfalfa. These results highlight the potential application of the visual reporter system for the stable transformation of alfalfa. Our improved CRISPR/Cas9 multiplex system enables convenient, high-efficiency multiplex genome editing in alfalfa, providing a versatile toolset to facilitate functional studies of multiple genes and gene families for basic research and the genetic improvement of alfalfa.

**Supplementary Information:**

The online version contains supplementary material available at 10.1007/s42994-025-00200-z.

## Introduction

The CRISPR (clustered regularly interspaced short palindromic repeats)/Cas9 (CRISPR-associated protein 9) system has been widely utilized for genome modification in plant species due to its simplicity, high efficiency, and versatility (Gao 2021; Li et al. 2024a). The editing efficiency of CRISPR/Cas9 is influenced by the expression levels of *Cas9* and single-guide RNA (sgRNA), which are generally driven by an RNA polymerase II (Pol II) promoter and a Pol III promoter (e.g., *U6* promoter), respectively. The use of endogenous *U6* promoters increases the editing efficiency of CRISPR/Cas9 in soybean (*Glycine max*), cotton (*Gossypium hirsutum*), maize (*Zea mays*), potato (*Solanum tuberosum*), grape, rubber tree, and sorghum (Dai et al. 2021; Johansen et al. 2019; Long et al. 2018; Massel et al. 2022; Qi et al. 2018; Ren et al. 2021; Sun et al. 2015). Multiplex genome editing was also studied for higher editing efficiency of a single gene or simultaneous editing of multiple genes. Generally, multiple sgRNAs can be produced by assembling several individual sgRNA expression cassettes, each transcribed from a separate Pol III promoter (Ma et al. 2015; Massel et al. 2022; Stuttmann et al. 2021; Wei et al. 2024; Xing et al. 2014). Multiple sgRNAs can also be expressed from a single transcript. Such polycistronic mRNAs are processed post-transcriptionally into individual sgRNAs by ribozymes, exogenous endoribonuclease (Csy4) or endogenous RNases (Čermák et al. 2017). Using the endogenous tRNA-processing system, polycistronic tRNA-sgRNA (PTG) driven by a single Pol III/Pol II promoter has been widely used in multiplex gene editing (Luo et al. 2021; Wang et al. 2018a; Xie et al. 2015; Zhou et al. 2023). tRNA might also function as a transcriptional enhancer for Pol III because it has internal promoter elements (box A and B) in its sequence that recruit the Pol III complex (Wang et al. 2018b; Xie et al. 2015).

Alfalfa (*Medicago sativa* L.), known as “the queen of forages”, is an important legume forage crop worldwide with high yield, high nutritional values, and wide adaptability. Breeding for the genetic improvement of alfalfa has been hindered due to its autotetraploid genome and self-incompatibility (Chen et al. 2020). To study gene functions or elucidate complex traits regulated by multiple genes, researchers must introduce multiple mutations into this plant simultaneously. Using CRISPR/Cas9-mediated multiplex editing to pyramid favorable alleles in an elite alfalfa variety will greatly accelerate the breeding process (Gao 2021; Luo et al. 2021). Therefore, developing highly efficient multiplex genome editing toolkits is important for both basic and applied research in alfalfa.

Only a few studies have focused on establishing and optimizing CRISPR/Cas9 systems in alfalfa. The first such system employed a single sgRNA driven by the *MtU6* promoter (Chen et al. 2020). Subsequently, the use of two sgRNAs driven by two *MtU6* promoters greatly increased the editing efficiency (Ye et al. 2022; Zheng et al. 2022). A PTG with four tRNA-sgRNA units driven by the *AtU6-26* promoter or a Pol II promoter was also used to improve the efficiency of this process (Wolabu et al. 2020, 2023, 2024; Zhao et al. 2024). Nevertheless, only a single gene was targeted in these studies, and endogenous alfalfa *U6* promoters have not yet been systemically identified and assessed. Highly active endogenous *U6* promoters must be identified to further improve CRISPR/Cas9-mediated multiplex editing in alfalfa.

Because stable transformation of alfalfa is inefficient and time-consuming, it is important to first identify efficient sgRNAs. The hairy root system has been employed for the rapid evaluation of gene editing systems and the screening of sgRNAs in several plant species, including soybean and pea (*Pisum sativum*) (Bai et al. 2019; Cheng et al. 2021; Li et al. 2022). A visual reporter allows for the rapid, convenient identification of transgene-positive hairy roots. Anthocyanin, the accumulation of which is induced by an R2R3 MYB transcription factor *OsC1*, has been used as a visible marker to detect the presence of transgenes in rice (*Oryza sativa*) (He et al. 2020b). RED HEART1 (RH1), an R2R3 MYB transcription factor that regulates anthocyanin-containing leaf markings in *Medicago truncatula* (Wang et al. 2021), could potentially be used as a visual reporter in alfalfa. Alternatively, the *RUBY* reporter, consisting of three genes encoding betalain biosynthetic enzymes, has been used for the visual selection of transgenic events in Arabidopsis (*Arabidopsis thaliana*), rice, cotton, tobacco, and *Plukenetia volubilis* (Ge et al. 2023; He et al. 2020a; Yu et al. 2023). Zhao et al. (2024) developed a rapid sgRNA screening system using hairy roots induced from alfalfa leaves incorporating the visible reporter *MtLAP1*, an R2R3 MYB transcription factor, which induces anthocyanin accumulation. However, there is still a need for a simple, rapid, high-efficiency hairy root system using visual reporters.

Here, we identified multiple highly active endogenous *U6* promoters in alfalfa and optimized the CRISPR/Cas9 multiplex system by combining multiple *MsU6* promoters with a PTG for the simultaneous editing of multiple genes in alfalfa, coupled with two different visual reporters. We also developed a simple hairy root system for alfalfa using germinated seeds, as explants, for the rapid evaluation of our gene editing system and to screen sgRNAs.

## Results

### Identification of endogenous alfalfa *U6* promoters with high transcriptional activity

To optimize the CRISPR/Cas9-based multiplex gene editing system, we systematically identified endogenous alfalfa *U6* promoters. BLAST searches using the *AtU6-26* sequence as a query revealed 15 (*MtU6.1* to *MtU6.15*) and 36 (*MsU6a* to *MsU6q*) *U6* genes in the model legume *M. truncatula* Jemalong A17 and the *Medicago sativa* CADL genome, respectively. Phylogenetic analysis and multiple sequence alignment revealed the conservation and variability of these *Medicago U6* promoters (Fig. S1; Fig. S2). All promoters contained the two conserved motifs essential for transcription, a TATA-like box, and an upstream sequence element (USE) (Fig. S2). To identify alfalfa *U6* promoters with high and stable transcriptional activity, we cloned these promoters from alfalfa cultivar Zhongmu No. 1 and used them to drive *GUS* reporter expression. We introduced the *MsU6::GUS* constructs into alfalfa leaves via *Agrobacterium* infiltration for transient expression. The promoters were categorized into three groups based on GUS staining: strong (dark blue staining), moderate (light blue staining), and weak promoters (faint staining/no staining) (Fig. S3). We selected the three strong promoters, *MsU6d1*, *MsU6g1* and *MsU6d3* promoters, to test their efficiencies for CRISPR/Cas9-mediated gene editing in alfalfa.

### Comparison of expression strength and gene editing efficiency of *MsU6* promoters with *AtU6*/*MtU6* promoters in the optimized alfalfa hairy root system

**Fig. 1:**
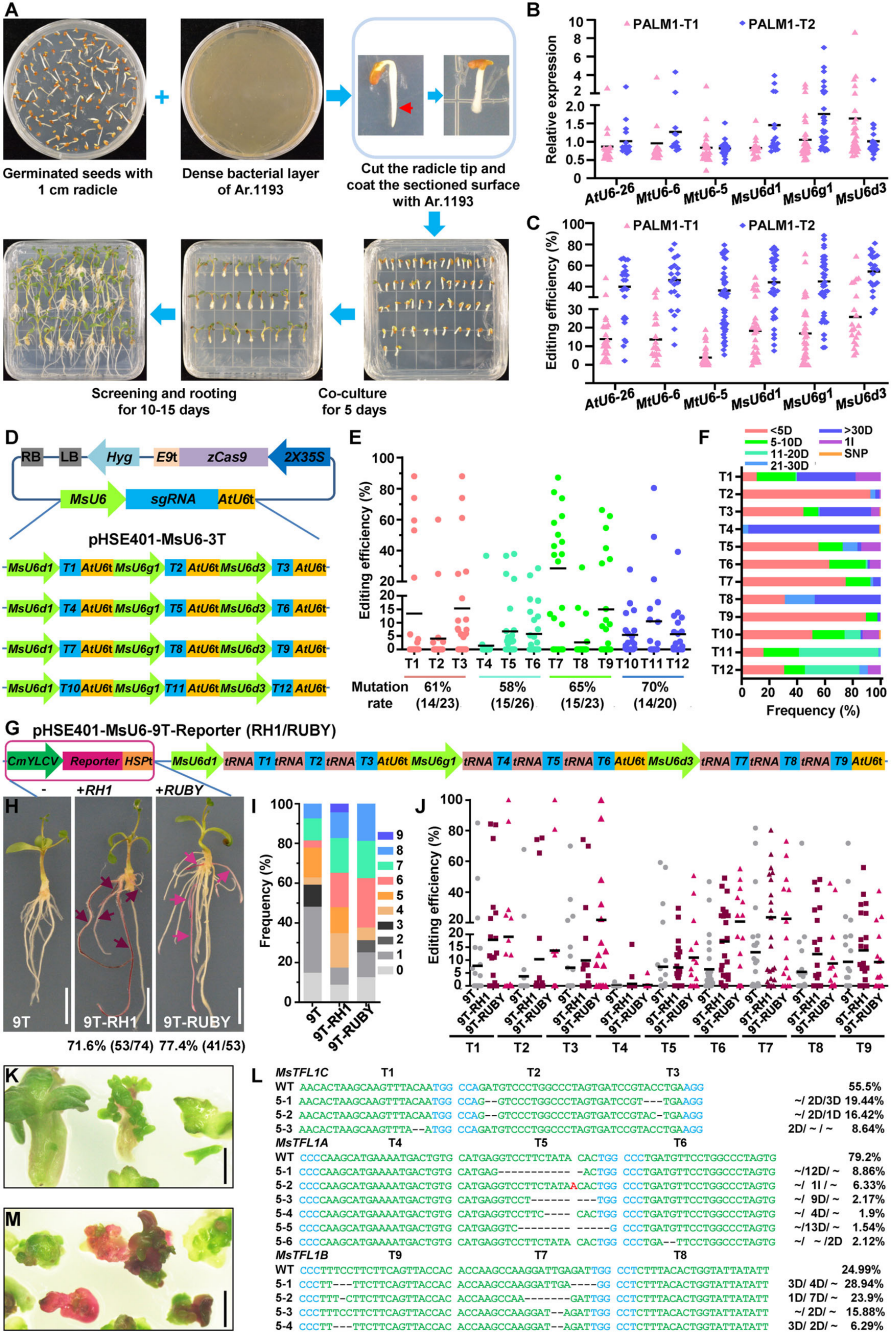
Efficient multiplex genome editing in alfalfa using an improved CRISPR/Cas9 multiplex system. **A** Workflow of *Agrobacterium rhizogenes*-mediated hairy root transformation for alfalfa. Comparison of relative expression level (**B**) and gene editing efficiency (**C**) of two sgRNAs driven by different *U6* promoters in alfalfa hairy root system. Quantitative RT-PCR was performed using specific target sequence and sgRNA scaffold as primers; Hygromycin-resistance gene was used as the reference control. Editing efficiency represents the percentage of reads with mutations. **D** Schematic diagrams of the four pHSE401-MsU6-3T vectors. T1–T3, T4–T6, T7–T9, and T10–T12 target *MsTFL1C*, *MsTFL1A*, *MsTFL1B*, and *MsBFT* respectively. **E** Mutation rates and editing efficiencies of individual target sites induced by the four pHSE401-MsU6-3T vectors. The mutation rate equals the ratio of hairy roots showing edited reads to the total number of hairy roots. The editing efficiency at a given target site represents the percentage of edited reads compared to the total number of reads in one sample. **F** Mutation types and frequencies at T1–T12 target sites induced by the four pHSE401-MsU6-3T vectors. D, deletion. I, insertion. SNP, substitution. **G** Schematic diagrams of pHSE401-MsU6-9T, -9T-RH1, and -9T-RUBY. **H** Representative photographs of hairy roots induced by 9T, 9T-RH1, and 9T-RUBY. Bars, 1 cm. **I** Frequencies of simultaneous editing of different numbers of target sites in hairy roots induced by 9T, 9T-RH1, and 9T-RUBY. **J** Editing efficiencies of individual target sites in hairy roots induced by 9T, 9T-RH1, and 9T-RUBY. **K** T_0_ regenerated shoots harboring 9T. Bars, 5 mm. **L** Representative mutations of 9T T_0_ regenerated shoots. Blue letters indicate the protospacer-adjacent motif (PAM) sequences, green letters indicate the sgRNA sequences, red letter indicates an insertion, WT, wild type. “D”, “I”, and “~” indicate deletion, insertion, and no editing, respectively. **M** Red T_0_ regenerated shoots harboring 9T-RUBY. Bars, 5 mm

To rapidly and efficiently evaluate the gene editing system and perform sgRNA screening, we firstly established an optimized hairy root system for alfalfa. Following infection, co-culture, screening and rooting, the induced hairy roots can be used for genotyping (Fig. 1A), which takes only ~ 2–3 weeks.

Then we replaced the *AtU6* promoters in the original CRISPR/Cas9 pHSE401 toolkit (Xing et al. 2014) with reported *MtU6* promoters (Ye et al. 2022) or our selected *MsU6* promoters. These pHSE401-AtU6/MtU6/MsU6 toolkits were applied to target alfalfa *Palmate-like pentafoliata1* (*MsPALM1*) gene with two sites (Fig. S4) in alfalfa hairy root system for comparison of expression level and gene editing efficiency of different *U6* promoters. Overall, all six *U6* promoters worked, but screened *MsU6* promoters exhibited a better performance in terms of editing efficiencies of two targets consistent with their relatively high expression strength (Fig. 1B, C). Editing efficiencies of both targets driven by *MtU6-5* promoter were the lowest may result from their low expression level, while *MtU6-6* promoter worked well in alfalfa, which may be due to the fact that the sequence of *MtU6-6* promoter shares a high degree of similarity with *MsU6g1* promoter, as shown in Fig. S1, *MtU6-6* is on the same branch with *MsU6g1* in the phylogenetic analysis. In addition, the high expression level of *MsU6d3*-driven T1 may contribute to its significantly increased editing efficiency (Fig. 1B, C).

### Multiplex genome editing mediated by pHSE401-MsU6-3T in the optimized alfalfa hairy root system

To further test the efficiency of pHSE401-MsU6 toolkit (Fig. S5) for multiplex genome editing in alfalfa, we selected 12 sgRNAs (T1-T12) targeting four genes of the *TFL1* family: *MsTFL1A*, *MsTFL1B*, *MsTFL1C*, and *MsBFT* (*BROTHER OF FT AND TFL1*) (Lorenzo et al. 2023; Fig. S6A), using three targets per gene (Fig. S6B). The four pHSE401-MsU6-3T vectors (Fig. 1D) constructed via PCR amplification and Golden Gate cloning were then introduced into Zhongmu No.1 hairy roots. One infected seedling usually produced 1 to 10 or more hairy roots, which were treated as one sample. Of the 23, 26, 23, and 20 samples containing each pHSE401-MsU6-3T construct, 14, 15, 15, and 14 were successfully edited, respectively, giving a mutation rate of 58–70% (Fig. 1E). Further analysis of percentage of edited reads per sample revealed the editing efficiency varied greatly among the 12 target sites: 13.4% (up to 88.0%) at T1, 4.1% (up to 60.1%) at T2, 15.5% (up to 88.0%) at T3, 1.5% (up to 36.6%) at T4, 6.8% (up to 37.8%) at T5, 5.7% (up to 28.6%) at T6, 28.5% (up to 87.2%) at T7, 2.7% (up to 29.2%) at T8, 15.0% (up to 66.2%) at T9, 5.4% (up to 27.8%) at T10, 10.5% (up to 80.4%) at T11, and 5.7% (up to 39.2%) at T12 (Fig. 1E). We detected three types of mutations at the 12 target sites, with the majority comprising deletions, followed by insertions and substitutions (Fig. 1F). All three *MsU6* promoters were effective for CRISPR/Cas9-mediated genome editing in alfalfa, and all 12 sgRNAs worked, though with different editing efficiencies, and the high variation of editing efficiency at each target site was perhaps due to the different ratios of transgene-positive hairy roots per sample.

### Efficient CRISPR/Cas9-mediated multiplex genome editing in alfalfa hairy roots using a visual reporter system

To further increase the efficiency of our CRISPR/Cas9 multiplex editing system, we combined the *MsU6* promoters with the endogenous tRNA-processing system (Xie et al. 2015), generating tandemly arrayed multiple *MsU6*-driven PTG expression cassettes. We added one tRNA sequence next to the *MsU6* promoter of each pHSE401-MsU6 toolkit vector, generating the pHSE401-MsU6-tRNA toolkit comprising pHSE401-MsU6d1-tRNA, pT-gtMsU6g1-tRNA, and pT-gtMsU6d3-tRNA (Fig. S7A). We also used the pGTR plasmid to amplify the PTG to construct the final multiplex vector. In addition, we used *MsU6g4*, *a* group II promoter, to generate one more expression cassette, the corresponding vector was pT-gtMsU6g4-tRNA (Fig. S7A). To facilitate the identification of transgenic events, we inserted the *RH1/RUBY* reporter unit driven by the *CmYLCV* promoter (Čermák et al. 2017) into pHSE401-MsU6d1-tRNA to produce anthocyanin or betalain, resulting in pHSE401-MsU6d1-tRNA-RH1/RUBY (Fig. S7B). Through PCR amplification and two rounds of Golden Gate cloning, the efficiency of sgRNA cassette assembly greatly increased. We ultimately constructed pHSE401-MsU6-9T (9T) targeting three genes (*MsTFL1A*, *MsTFL1B*, and *MsTFL1C*) with or without *RH1*/*RUBY*, respectively (Fig. 1G). To test whether *CmYLCV::RH1/RUBY* can produce anthocyanin or betalain in plants, we infiltrated *Nicotiana benthamiana* leaves with *Agrobacterium* carrying pHSE401-MsU6d1-tRNA-RH1/RUBY or 9T-RH1/RUBY. The transient expression of *CmYLCV::RH1/RUBY* led to the production of anthocyanin or betalain in *Nicotiana benthamiana* leaves (Fig. S8). Given the relatively low editing efficiencies of all three targets (T4-T6) for *MsTFL1A*, we selected two new target sites for this gene (T13 and T14) (Fig. S6B). After removing the target site of each *TFL1* gene with the lowest editing efficiency (T2 for *MsTFL1C*, T4 for *MsTFL1A*, and T8 for *MsTFL1B*), 12T, 12T-RH1, and 12T-RUBY targeting four genes (*MsTFL1A*, *MsTFL1B*, *MsTFL1C* and *MsBFT*) were constructed, containing 11 target sites with T13, T14, and two T6 targets (Fig. S9A).

When we introduced the six constructs into alfalfa hairy roots, 71.6% (53/74) of 9T-RH1 and 77.4% (41/53) of 9T-RUBY transformed seedlings produced hairy roots containing anthocyanin and betalain, respectively, which were easily identified by their purple or red color. In general, transformation with the *RH1* reporter produced a higher percentage of colored hairy roots induced from the same seedling than *RUBY* (Fig. 1H). We subjected colored roots transformed with 9T-RH1/RUBY or 12T-RH1/RUBY constructs to Hi-TOM sequencing. All six constructs successfully induced multiplex genome editing. The use of the visual reporter RH1/RUBY allowed us to detect a higher percentage of more edited target sites simultaneously. Whereas in 77.8% of 9T transformed hairy roots, no more than five target sites were edited simultaneously (14.8% [0], 33.3% [1], 11.1% [3], 3.7% [4], 14.8% [5]), in 52.2% and 62.5% of 9T-RH1 and 9T-RUBY transformed hairy roots, at least six target sites were edited simultaneously, respectively. For 9T, 9T-RH1, and 9T-RUBY transformed hairy roots, 7.4%, 13.0%, and 18.8% contained eight target sites that were edited simultaneously, respectively. Notably, nine target sites were edited simultaneously in 4.4% of 9T-RH1 transformed hairy roots (Fig. 1I). Similarly, 75.9% (63/83) and 79.7% (59/74) of 12T-RH1 and 12T-RUBY transformed hairy roots accumulated anthocyanin or betalain, and 66.7% of 12T transformed hairy roots contained no more than six target sites edited simultaneously, while 54.6% and 64.3% of 12T-RH1 and 12T-RUBY transformed hairy roots had at least seven target sites edited simultaneously, respectively. Finally, 23.8%, 18.2%, and 21.4% of 12T, 12T-RH1, and 12T-RUBY transformed hairy roots, respectively, contained at least nine target sites that were edited simultaneously (Fig. S9B).

The average editing efficiencies of 9T were 7.8% (up to 84.9%) at T1, 3.8% (up to 67.7%) at T2, 7.1% (up to 69.9%) at T3, 0.1% (up to 1.5%) at T4, 7.4% (up to 59.1%) at T5, 6.4% (up to 46.8%) at T6, 13.1% (up to 81.6%) at T7, 5.4% (up to 71.8%) at T8, and 9.3% (up to 71.8%) at T9. Compared to 9T, 9T-RH1 and 9T-RUBY exhibited a 2.7 fold (up to ninefold) and 2.4 fold (up to 3.6 fold) higher average editing efficiency at a given target site, respectively (Fig. 1J). The use of *RH1/RUBY* allowed us to detect higher editing efficiencies at a given target site in more samples, highlighting the efficiency of screening positive transgenic hairy roots using the visual reporter. The editing efficiencies of 12T were 7.2% (up to 34.5%) at T1, 1.9% (up to 17.2%) at T3, 8.0% (up to 53.5%) at T5, 8.7% (up to 81.86%) at T6, 12.4% (up to 75.7%) at T7, 11.2% (up to 74.1%) at T9, 0.2% (up to 2.19%) at T10, 3.8% (up to 26.6%) at T11, 6.8% (up to 50.5%) at T12, 5.7% (up to 55.1%) at T13, and 7.1% (up to 55.3%) at T14. Compared to 12T, 12T-RH1 and 12T-RUBY exhibited a 1.3 fold (up to 2.6 fold) and 1.9 fold (up to 11 fold) higher average editing efficiency at a given target site, respectively, with no increase or a small decline at some target sites (Fig. S9C).

### Use of the improved CRISPR/Cas9 multiplex system for the stable transformation of alfalfa

To investigate the efficiency of 9T in generating mutations in alfalfa, we introduced this construct into alfalfa by *Agrobacterium*-mediated transformation and selected regenerated shoots for the rapid identification of mutations (Fig. 1K). Six out of 23 samples were transgene-positive based on PCR. Hi-TOM sequencing revealed that four samples were edited, with 2–7 target sites edited simultaneously (Table S1). The editing efficiencies of sample #5 with 7 target sites edited simultaneously were 8.6% at T1, 35.9% at T2 and T3, 20.8% at T5, 2.12% at T6, 75% at T7, and 59.1% at T9, whereas no mutations were observed at T4 or T8 (Fig. 1L), which is consistent with their lower editing efficiencies in the hairy root system. When we introduced 9T-RUBY into alfalfa, we observed red coloration after the calli were transferred onto regeneration medium, followed by the appearance of red regenerated shoots (Fig. 1M). Together, these results highlight the feasibility of using the pHSE401-MsU6-tRNA toolkit for multiplex gene editing of alfalfa plants and the potential application of visual reporters for screening stable transgenic events in alfalfa.

## Discussion

CRISPR/Cas9-mediated genome editing is highly useful for biological research and crop improvement, but its widespread application in alfalfa has been impeded by the lack of suitable Pol III promoters and an efficient multiplex editing system. Here, we identified multiple highly active alfalfa endogenous *U6* promoters and demonstrated the efficacy of the three most active promoters for genome editing using an optimized hairy root system. We then developed an optimized CRISPR/Cas9 multiplex system containing 3 or 4 tandemly arrayed *MsU6*-promoter-driven PTG expression cassettes for the simultaneous editing of 3 or 4 genes, incorporating the visual reporter *RH1* or *RUBY*. This toolkit is efficient for multiplex editing in the visual hairy root system and the stably transformed regenerated shoots. Furthermore, the red-colored regenerated shoots suggested the potential of the visual reporter for application in alfalfa stable transformation.

Mutations were previously introduced at all eight sites of four homologous rice *MITOGEN-ACTIVATED PROTEIN KINASE* genes by designing a PTG gene encoding eight sgRNAs (Xie et al. 2015). High-efficiency multiplex editing of the *Hd1* promoter was recently achieved using eight tRNA-sgRNA units driven by a single *U3* promoter (Zhou et al. 2023). Accordingly, our improved multiplex genome editing system, containing four *MsU6*-promoter-driven PTG expression cassettes, has great potential for highly efficient multiplex genome editing to dissect complex traits involving multiple genes and for the stacking of superior traits in alfalfa. However, as the number of sgRNAs increases, the availability of Cas9 nuclease may become a limiting factor for higher-order multiplex editing (Stuttmann et al. 2021). To increase *Cas9* expression, the *35S* promoter could be replaced by strong endogenous promoters, such as the Arabidopsis *UBIQUITIN10* promoter (Wolabu et al. 2020) or the soybean *elongation factor 1A* promoter *proGmScreamM4* (*pM4*) (Bai et al. 2020). Strong endogenous promoters from alfalfa could also be identified and used to drive *Cas9* expression in future studies.

The *RUBY* reporter was recently used to stably transform soybean, facilitating the visual selection of genome-edited but transgene-free soybean seedlings (Chen et al. 2024). We observed red regenerated shoots during the stable transformation of alfalfa with 9T-RUBY, highlighting the potential of this reporter for genome editing in alfalfa, although the efficacy and accuracy of *RUBY* as a visual indicator of transformation and the presence of the transgene must be further explored. Moreover, simultaneous gene knockout and gene overexpression are often needed to obtain the desired genotype. A high-yielding, multi-resistant rice variety was recently developed using multigene transformation and gene editing (Li et al. 2024b). Our system, coupling the CRISPR/Cas9 cassette with a *RUBY* expression unit consisting of three genes, provides a promising strategy for simultaneous multigene knockout and multigene overexpression in alfalfa.

In summary, we developed an improved CRISPR/Cas9 system for the simultaneous editing of four genes in alfalfa by combining multiple endogenous *MsU6* promoters with the PTG strategy. This convenient, efficient multiplex gene editing system using visual reporters, coupled with the simple, rapid hairy root system, will greatly facilitate functional genomics studies and the genetic improvement of alfalfa and other legume crops.

## Materials and methods

### Plant materials and growth conditions

The *M. truncatula* ecotype R108 and alfalfa cultivar Zhongmu No. 1 were used in this study. Scarified R108 seeds were germinated overnight in moist Petri dishes and Zhongmu No. 1 clones were produced by stem cutting. Plants were grown in a greenhouse at 60–70% relative humidity under a 16-h light (24 °C)/8-h dark (22 °C) photoperiod. Surface-sterilized Zhongmu No. 1 seeds were germinated on 1/2 Murashige & Skoog (MS) medium (2.215 g L^−1^ MS basal medium with vitamins, 15 g L^−1^ sucrose, 7 g L^−1^ agar, pH 5.85) at 25 °C in the dark for 36 h.

### Sequence alignment and phylogenetic analysis

*Arabidopsis AtU6–26* (AT3G13855) was used as a query to identify *Medicago U6* genes from the Medicago Analysis Portal (https://medicago.legumeinfo.org/). The TERMINAL FLOWER 1 (TFL1) protein sequences of alfalfa, *M. truncatula*, soybean, *Lotus japonicus*, common bean (*Phaseolus vulgaris*), pea, Arabidopsis, and citrus (*Citrus sinensis*) were selected from SequenceServer v3.1.3 (https://sequenceserver.legumeinfo.org/) and Phytozome v13 (https://phytozome-next.jgi.doe.gov/). Sequence alignment was performed using Clustal W, and phylogenic analysis was performed using MEGA5.0 (neighbor-joining method using default settings with 1000 bootstrap replications).

### *MsU6* promoter cloning and construction of *MsU6::GUS*

The *GUS* (*β-glucuronidase*) gene was amplified from the pMDC162 vector and cloned into the linearized pMDC32 vector digested with *Kpn*I and *Pac*I through homologous recombination (HR) with a Uniclone One Step Seamless Cloning Kit (Genesand) to generate pMDC32-GUS. To construct *MsU6::GUS* vectors, the regions approximately 0.6–1 kb upstream of the transcription start sites of *MsU6* genes were amplified from Zhongmu No. 1 genomic DNA as promoter regions and cloned into pMDC32-GUS instead of the 35S promoter via the *Hin*dIII and *Kpn*I sites through HR. All primers used in this study are listed in Table S2.

### Vector optimization for alfalfa genome editing

To construct the pHSE401-MtU6/MsU6 toolkit, the ~ 0.5 kb *MtU6-6*/*MtU6-5* promoter or *MsU6d1*/*g1*/*d3*/*g4* promoter was amplified from R108 genomic DNA or the corresponding *MsU6::GUS* vector respectively and fused with the SpR-sgRNA scaffold-AtU6 terminator fragment amplified from pHSE401 (Xing et al. 2014) by overlapping PCR. The resulting fragment was cloned into the *Hin*dIII-digested pHSE401 vector through HR, generating pHSE401-MtU6/MsU6 with the original *AtU6-26* promoter replaced by the *MtU6*/*MsU6* promoter. The pT-gtAtU6 or pT-gtMtU6 or pT-gtMsU6 vector was constructed by fusing the sgRNA-scaffold-AtU6t fragment amplified from the pCBC vector (Xing et al. 2014) with the *AtU6-26* or *MtU6-6*/*MtU6-5* or *MsU6d1/g1*/*d3*/g4 promoter with the original *Bsa*I site mutated and ligating the resulting fragment into the pEASY®-Blunt Zero Cloning vector.

To construct the pHSE401-MsU6-tRNA toolkit, the tRNA fragment amplified from pGTR (Xie et al. 2015) was fused with the SpR fragment amplified from pHSE401-MsU6d1 by overlapping PCR, and the resulting fragment was ligated into the *Bsa*I-digested pHSE401-MsU6d1 vector through HR, generating pHSE401-MsU6d1-tRNA. The pT-gtMsU6g1/d3/g4-tRNA vector was constructed by fusing the sgRNA-scaffold-AtU6t-MsU6g1/d3/g4 fragment amplified from the pT-gtMsU6g1/d3/g4 vector with the tRNA fragment and ligating the resulting fragment into the pEASY^®^-Blunt Zero Cloning vector.

To construct the pHSE401-MsU6-tRNA-reporter toolkit, *RH1* was amplified from pEarleyGate203-RH1 (Wang et al. 2021), *RUBY* was amplified pC304 (Wang et al. 2023), and the *CmYLCV* promoter and *Heat Shock Protein* (*HSP*) terminator were amplified from pDIRETCT_22C (Čermák et al. 2017). The *CmYLCV* promoter, *RH1*/*RUBY*, and *HSP* terminator fragments were fused by overlapping PCR, generating the CmYLCV::RH1/RUBY-HSPt fragment, which was cloned into the *Pme*I site of the pHSE401-MsU6d1-tRNA vector.

### CRISPR vector construction

The target sites for sgRNAs of *MsPALM1* and *MsTFL1* family genes were designed using CRISPR-P 2.0 (http://crispr.hzau.edu.cn/CRISPR2/). Based on Xing et al. (2014) and Xie et al. (2015), the PALM1-sgRNA module amplified using the pT-gtAtU6/MtU6/MsU6 vector as a PCR template was assembled into the corresponding pHSE401-AtU6/MtU6/MsU6 via the Golden Gate cloning for comparison of expression strength and gene editing efficiency of different U6 promoters. To construct pHSE401-MsU6-3T, the pT-gtMsU6g1/d3 vectors were used as PCR templates to amplify the sgRNA modules, and the modules were assembled into pHSE401-MsU6d1 via the Golden Gate cloning. To construct pHSE401-MsU6-9T/12T, pT-gtMsU6g1/d3/g4-tRNA, coupled with the pGTR vector, were used as PCR templates to amplify the tRNA-sgRNA modules, and the modules were assembled into pHSE401-MsU6d1-tRNA or the pHSE401-MsU6d1-tRNA-reporter vector via two rounds of Golden Gate cloning.

### Plant transformation

For transient expression, *Agrobacterium tumefaciens* strain AGL1 cells carrying *MsU6::GUS* vectors or CRISPR vectors with *CmYLCV::RH1*/*RUBY* were introduced into *Nicotiana benthamiana* or alfalfa leaves via *Agrobacterium*-mediated infiltration, respectively. GUS staining was performed as described previously (Niu et al. 2015), and images of stained alfalfa leaves were collected with a digital camera mounted on an Olympus SZX-16 stereoscope.

The alfalfa hairy root system was developed based on the protocol for *Agrobacterium rhizogenes*-mediated transformation of *Medicago truncatula* (Boisson-Dernier et al. 2001), with some modifications. Briefly, germinated alfalfa seeds were inoculated with *A. rhizogenes* strain Ar.1193. After ~ 36 h of germination, 1 cm long radicles from seedlings were cut approximately 3 mm from the root tip, and the freshly cut surfaces were inoculated with *A. rhizogenes* carrying the corresponding construct by lightly scraping the surface of the dense bacterial layer. The infected seedlings were placed on square plates (10 cm × 10 cm) with 1/2 MS medium containing 100 µM acetosyringone for co-culture in the greenhouse. Five days later, the incision area swelled, from which hairy roots began to emerge. The seedlings were transferred to 1/2 MS medium containing 5 mg L^−1^ hygromycin B (Sigma) and 100 mg L^−1^ cefotaxime (PhytoTech) and cultured for 10–15 days for screening and rooting.

For stable transformation, pHSE401-MsU6-9T and pHSE401-MsU6-9T-RUBY was introduced into alfalfa via *Agrobacterium*-mediated transformation as described previously (Fu et al. 2015).

### Gene expression analysis

Total RNA was extracted from hairy roots using TRIzol Reagent (Invitrogen) and reverse-transcribed using HiScript III 1st Strand cDNA Synthesis Kit (Vazyme) with a mixture of reverse primers specific to the Hyg gene and sgRNA. Quantitative RT-PCR was performed using Taq Pro Universal SYBR qPCR Master Mix (Vazyme). The results of target sgRNA expression were quantified and normalized relative to the reference control (*Hyg*) using the comparative CT method. The values presented in the figure were calculated from three technical replicates.

### Detection of on-target editing in hairy roots and T_0_ regenerated shoots

Genomic DNA was extracted from hairy roots and T_0_ regenerated shoots using the cetyltrimethylammonium bromide (CTAB) method. Genomic regions flanking sgRNA target sites were amplified using specific primers. The resulting PCR products were subjected to next-generation sequencing (NGS) using the Hi-TOM platform (Sun et al. 2024).

## Supplementary Information

Below is the link to the electronic supplementary material.

Supplementary file1 (PDF 1038 KB)

## Data Availability

All data generated or analyzed during this study are available from the corresponding author upon reasonable request.
