# Supplementary material for: Convenient, high-efficiency multiplex genome editing in autotetraploid alfalfa using endogenous U6 promoters and visual reporters
Source: aBIOTECH. 2025 Feb 10;6(1):81–90. doi: 10.1007/s42994-025-00200-z (PMC11889276; doi:10.1007/s42994-025-00200-z)
Supplement: Supplementary file 1 — Supplementary file1 (PDF 1038 KB) [file 42994_2025_200_MOESM1_ESM.pdf]

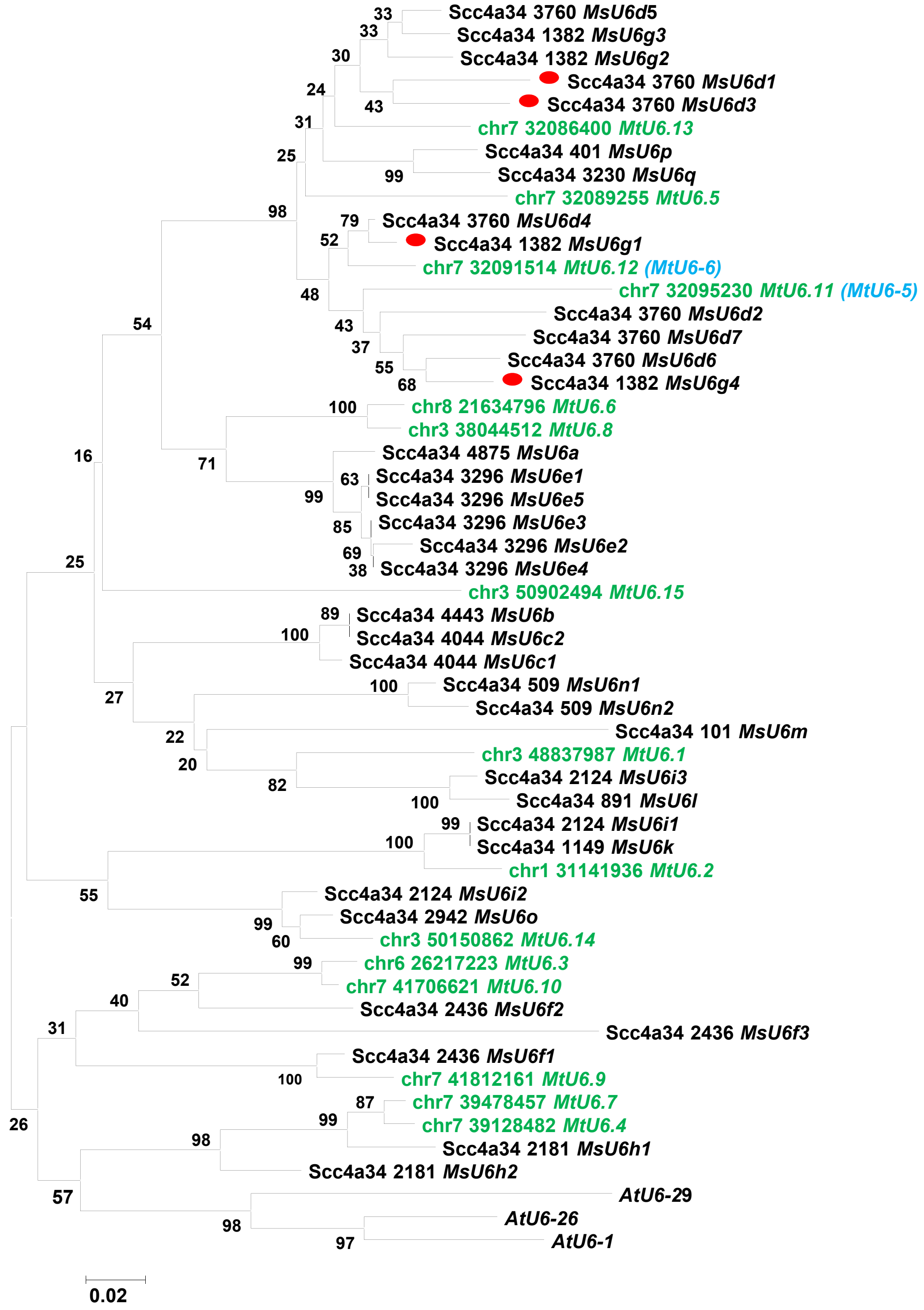

Fig S1. Phylogenetic analysis of *U6* snRNA genes and promoters in alfalfa (*Ms*), *M.truncatua* (*Mt*), and Arabidopsis (*At*).

MsU6 genes located on different scaffolds were named with different lowercase letters, while those located on the same scaffold were further distinguished with numbers. *U6* snRNA genes and 500 bp upstream of the transcription start site promoter sequences were aligned using Clustal W, and a neighbour-joining phylogenetic tree was constructed using MEGA5 software. Numbers on branches indicate bootstrap percentages for 1000 replicates.

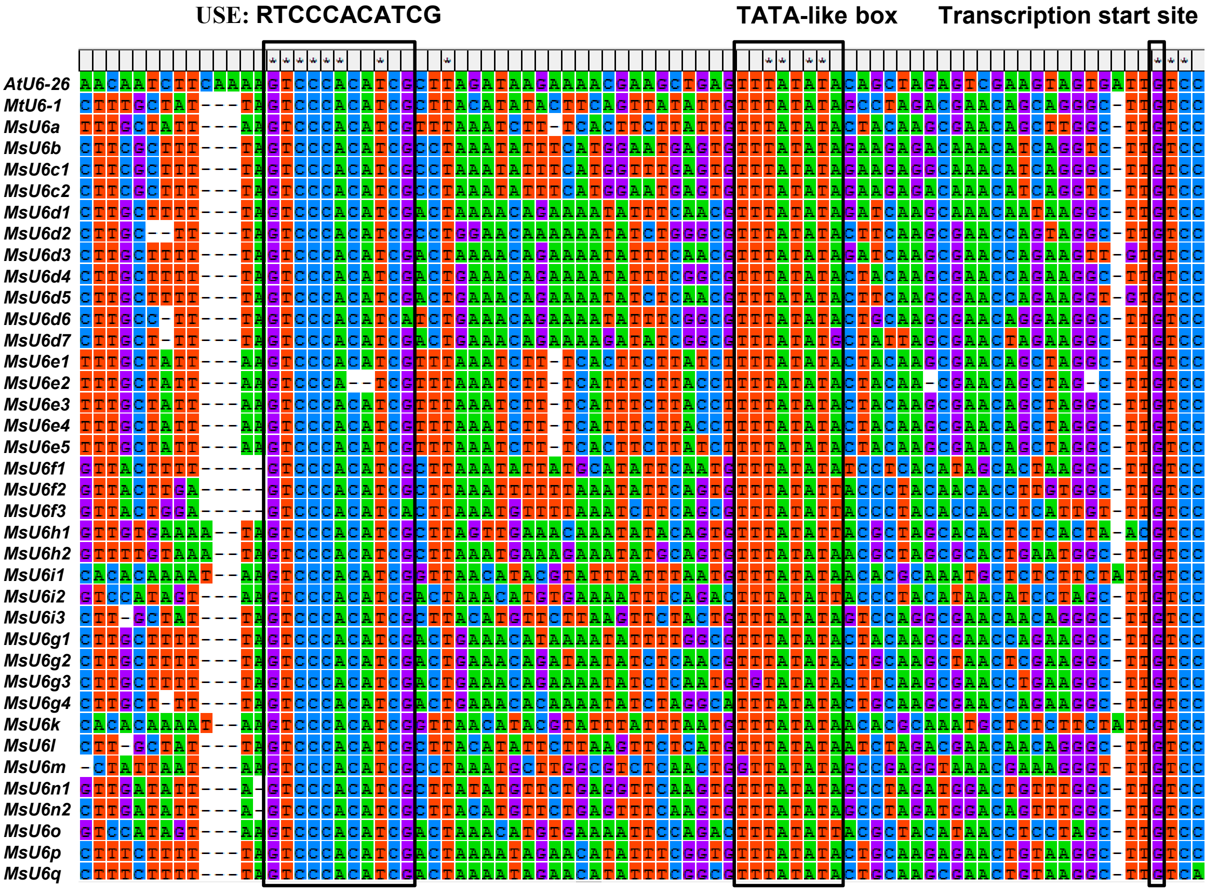

Fig S2. Analysis of alfalfa *MsU6* promoter sequences.

Multiple alignments of alfalfa, *M.truncatula*, and Arabidopsis *U6* promoter sequences. Upstream Sequence Elements (USE), the TATA-like boxes, and the transcription start sites of the U6 small nuclear (snRNA) transcripts are marked with black boxes.

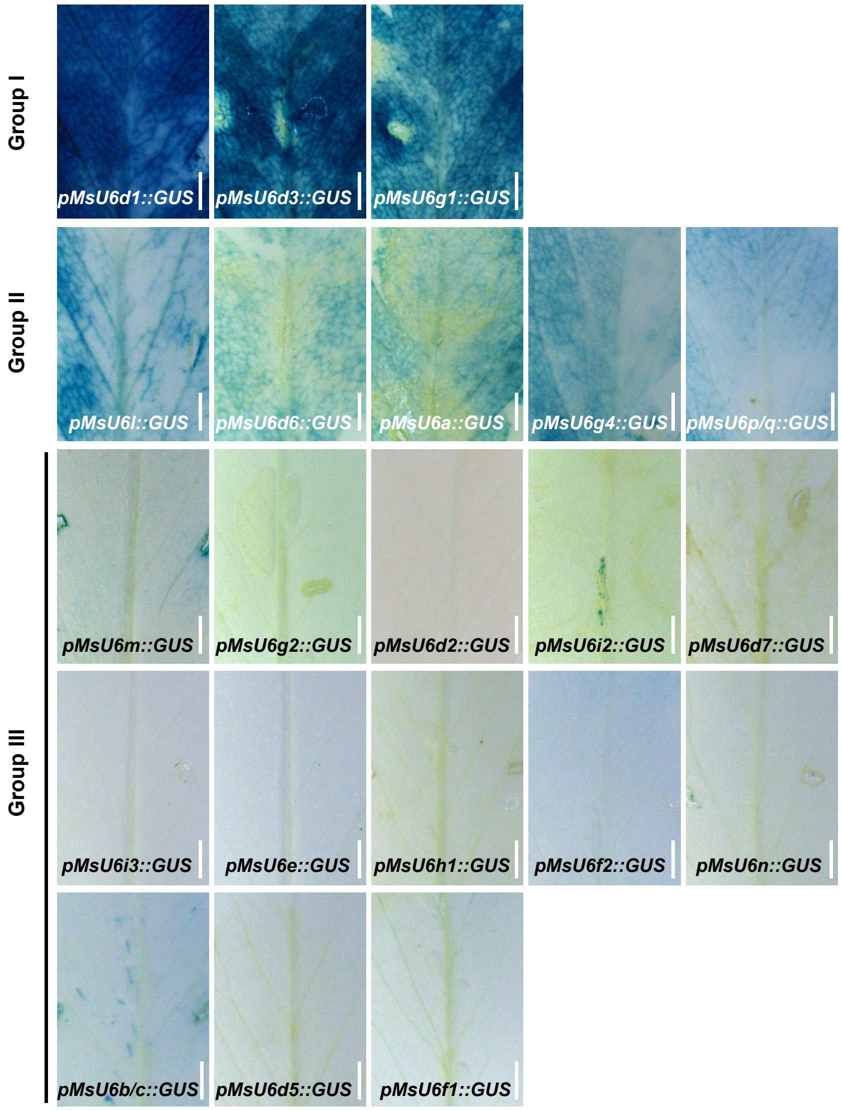

Fig S3. GUS staining in alfalfa leaves infiltrated with *Agrobacterium* carrying different promoter constructs.

600-1000 bp upstream of the transcription start site sequences were cloned from alfalfa Zhongmu NO.1 as *MsU6* promoters to drive *GUS (β-glucuronidase*). These promoters were divided into three groups according to the GUS staining results. Bars, 1 mm.

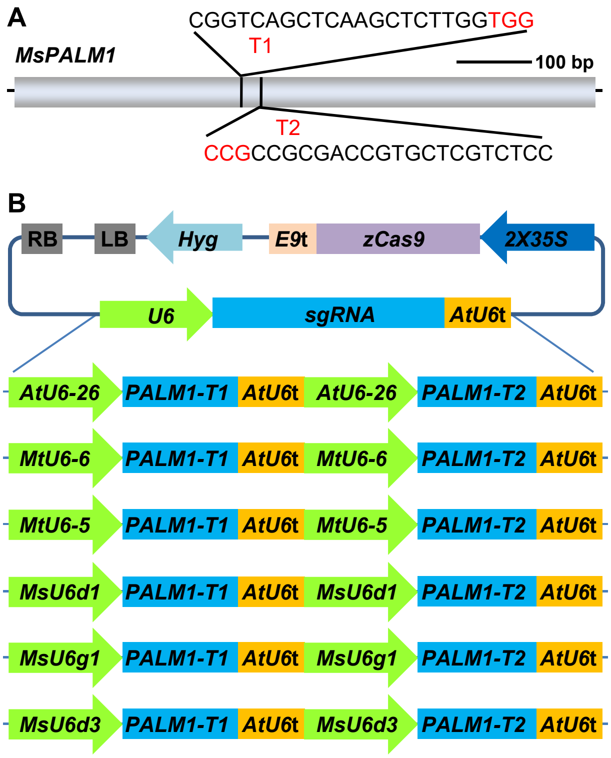

Fig S4. Schematic diagrams of pHSE401-2T driven by different *U6* promoters.

A Schematic representation of *MsPALM1* gene structure, showing target sites for sgRNAs. Boxes represent exons. PAM was labeled by red color. B Schematic diagrams of pHSE401-2T driven by different *U6* promoters, including *AtU6*, *MtU6* and *MsU6* promoters.

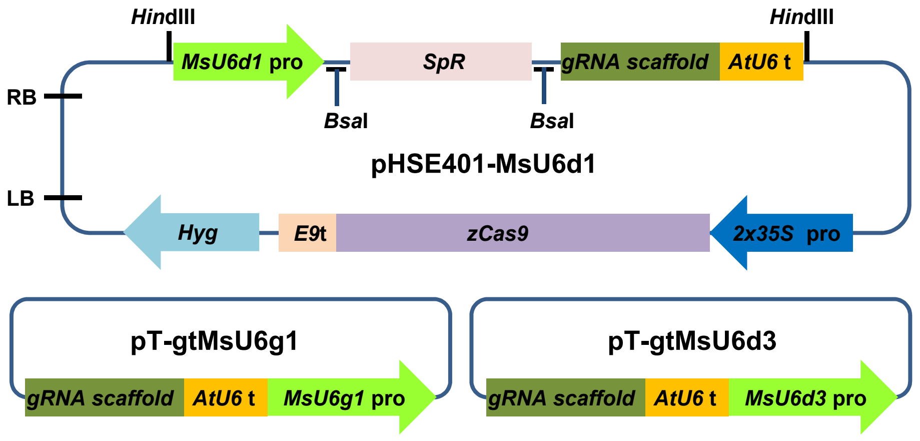

Fig S5. Schematic diagrams of vectors of pHSE401-MsU6 toolkit.

The original *AtU6-26* promoter of pHSE401 was replaced with *MsU6d1* promoter, generating pHSE401-MsU6d1. *Zea mays* codon-modified zCas9 was driven by the *2×35S* promoter. The hygromycin resistance gene *Hyg* was used as a selectable marker for plant transformation. The pT-gtMsU6g1 and pT-gtMsU6d3 vector were used as PCR template to amplify the sgRNA modules, and the modules were assembled into pHSE401-MsU6d1 vector via the Golden Gate cloning. The pT-gtMsU6g1/pT-gtMsU6d3 vector was constructed by fusing *MsU6g1/MsU6d3* promoter to gRNA-scaffold-AtU6t fragment cloned from pCBC and ligating the whole fragment into *pEASY*®-Blunt Zero Cloning vector.

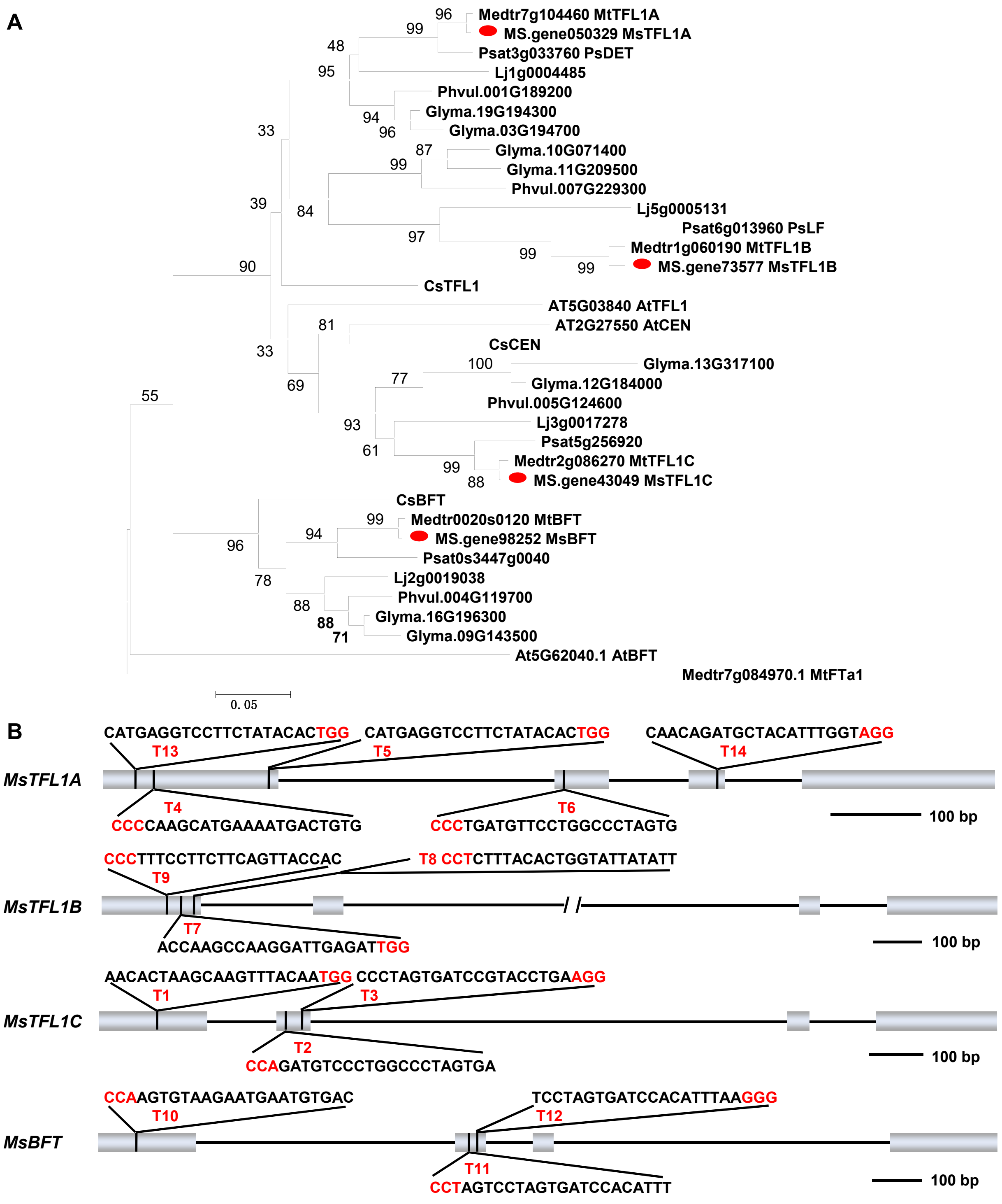

Fig S6. Phylogenetic analysis of TFL1 family proteins and sgRNA target sites of the selected four alfalfa genes.

A Phylogenetic analysis of TFL in alfalfa (Ms), *M. truncatula* (Medtr or Mt), Arabidopsis (At), soybean (Glyma), common bean (Phvul), *Lotus japonicus* (Lj), Pea (Psat) and Citrus (Cs). MsTFL1A, MsTFL1B, MsTFL1C and MsBFT are highlighted with red dots. Full-length protein sequences were aligned using Clustal W, and a neighbour-joining phylogenetic tree was constructed using MEGA5 software. Numbers on branches indicate bootstrap percentages for 1000 replicates. **B** Schematic representations of *MsTFL1A*, *MsTFL1B*, *MsTFL1C* and *MsBFT* gene structures, showing target sites for sgRNAs. Boxes represent exons and lines represent introns. PAM was labeled by red color.

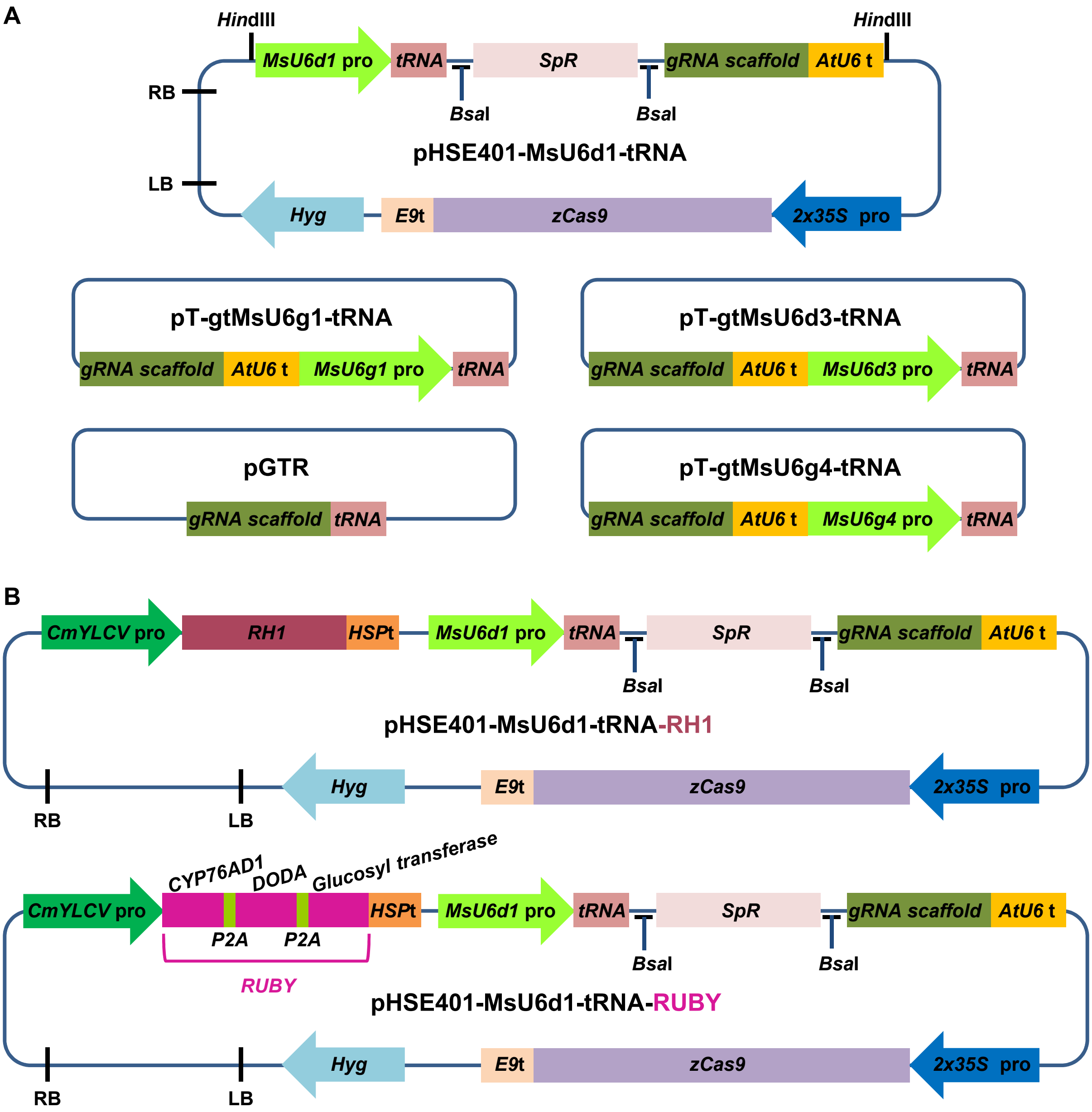

Fig S7. Schematic diagrams of vectors of pHSE401-MsU6-tRNA toolkit and pHSE401-MsU6-tRNA-reporter toolkit.

A The pHSE401-MsU6-tRNA toolkit was generated by adding the tRNA sequence next to the *MsU6* promoter of the pHSE401-MsU6 toolkit vectors respectively. The pT-gtMsU6g1-tRNA and pT-gtMsU6d3-tRNA vector, coupled with pGTR and pT-gtMsU6g4-tRNA, were used as PCR template to amplify the tRNA-sgRNA modules, and the modules were assembled into pHSE401-MsU6d1-tRNA vector via the Golden Gate cloning. **B** The pHSE401-MsU6-tRNA-reporter toolkit was constructed by inserting the *RH1*/*RUBY* reporter driven by the *CmYLCV* promoter into pHSE401-MsU6d1-tRNA vector. The *RUBY* reporter consists of three betalain biosynthetic genes *CYP76AD1*, *DODA* and *glucosyl transferase* linked with ‘self-cleaving’ 2A peptides.

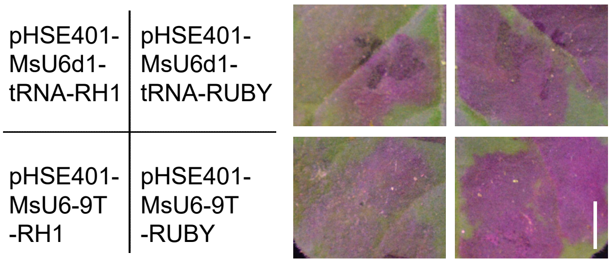

Fig S8. Transient expression of *RH1*/*RUBY* driven by the *CmYLCV* promoter in *Nicotiana benthamiana* leaves.

Agrobacterium carrying the expression cassette of *RH1*/*RUBY* under the control of *CmYLCV* promoter was infiltrated into *Nicotiana benthamiana* leaves. Pictures were taken 7 days after infiltration. Bars, 5 mm.

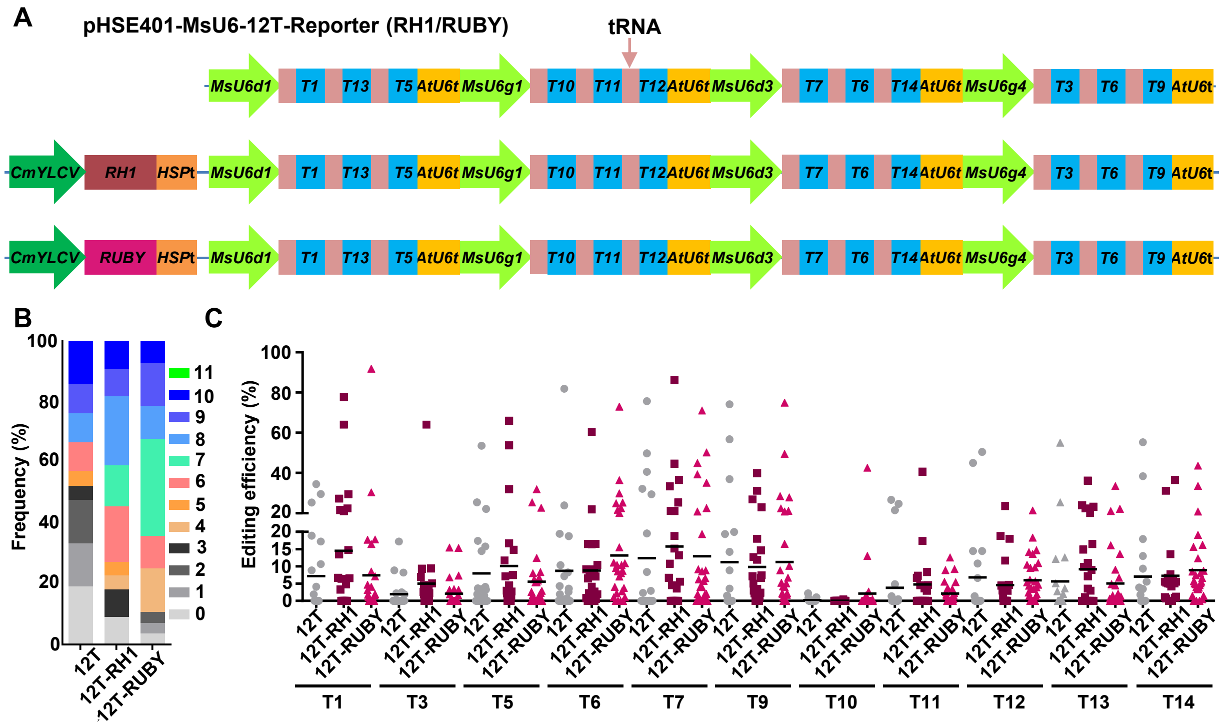

Fig S9. Multiplex gene editing in hairy root system induced by pHSE401-MsU6-12T, -12T-RH1 and -12T-RUBY.

A Schematic diagrams of pHSE401-MsU6-12T, -12T-RH1 and -12T-RUBY. **B** Frequencies of simultaneous editing of different numbers of target sites in hairy root system induced by 12T, 12T-RH1 and 12T-RUBY. **C** Editing efficiencies of individual target sites in hairy root system induced by 12T, 12T-RH1 and 12T-RUBY.

Table S1. Multiplex gene editing in T_0 regenerated shoots of 9T

No of tested regenerated shoots	No of positive regenerated shoots	No of edited regenerated shoots	No of simultaneous editing target sites
23	6	4	5 (#1)<br>4 (#2)<br>2 (#4)<br>7 (#5)

Table S2. Primers used in this study

Primer Name	Sequence (5'-3')
**For *pMsU6::GUS***	
GUS-MDC32-KpnI-F	cgactctagaggatccccgggtaccATGTTACGTCCTGTAGAAACC
GUS-MDC32-PacI-R	ggccgctctagaactagttaattaaTCATTGTTTGCCTCCCTGCTGCG
pMsU6a-GUS-MDC32-HindIII-F	gtaaaacgacggccagtgccaagcttGTGGATTTAGTACACCGCAACC
pMsU6e-GUS-MDC32-HindIII-F	gtaaaacgacggccagtgccaagcttAACTGCACCATCCATTTACTTG
pMsU6ae-GUS-MDC32-KpnI-R	gtttctacaggacgtaacatggtaccAAGCCTAGCTGTTCGCTTGTAG
pMsU6bc-GUS-MDC32-HindIII-F	gtaaaacgacggccagtgccaagcttCAATCCAATAGCAAGGGACGTA
pMsU6bc-GUS-MDC32-KpnI-R	gtttctacaggacgtaacatggtaccAAGMCCTGATGTTTGTCTCTTC
pMsU6d1d5-GUS-MDC32-HindIII-F	gtaaaacgacggccagtgccaagcttGATCACCAAGCAGCTAACCC
pMsU6d1-GUS-MDC32-KpnI-R	gtttctacaggacgtaacatggtaccAAGCCTTATTGTTTGCTTGATC
pMsU6d2-GUS-MDC32-HindIII-F	gtaaaacgacggccagtgccaagcttGCATATGGCAATGTTCATGTCT
pMsU6d2-GUS-MDC32-KpnI-R	gtttctacaggacgtaacatggtaccAAGCCTACTGGTTCGCTTGAAG
pMsU6d3g3pq-GUS-MDC32-HindIII-F	gtaaaacgacggccagtgccaagcttTTGGTCATTGTATCAAGCCTGTA
pMsU6d3-GUS-MDC32-KpnI-R	gtttctacaggacgtaacatggtaccACAACTTCTGGTTCGCTTGATC
pMsU6d5g3-GUS-MDC32-KpnI-R	gtttctacaggacgtaacatggtaccAAGCCTTCAGGTTCGCTTGAAG
pMsU6pq-GUS-MDC32-KpnI-R	gtttctacaggacgtaacatggtaccAAGCCTTACAGTTCTCTTGCAG
pMsU6d7-GUS-MDC32-HindIII-F	gtaaaacgacggccagtgccaagcttAAAGAAATCTAGCCTACGAGGA
pMsU6d7-GUS-MDC32-KpnI-R	gtttctacaggacgtaacatggtaccAAGCCTTCTAGTTCGCTAATAGC
pMsU6f1-GUS-MDC32-HindIII-F	gtaaaacgacggccagtgccaagcttAAACAATGATGGGTTGGTGC
pMsU6f1-GUS-MDC32-KpnI-R	gtttctacaggacgtaacatggtaccAAGCCTTAGTGCTATGTGAGGA
pMsU6f2-GUS-MDC32-HindIII-F	gtaaaacgacggccagtgccaagcttTATTGATGTGATGACCCAAATG
pMsU6f2-GUS-MDC32-KpnI-R	gtttctacaggacgtaacatggtaccAAGCCACAAGGTGTTGTAGGG
pMsU6g1-GUS-MDC32-HindIII-F	gtaaaacgacggccagtgccaagcttGCACTAGGTGTCATGCTATGTT
pMsU6g1-GUS-MDC32-KpnI-R	gtttctacaggacgtaacatggtaccAAGCCTTCTGGTTCGCTTGTAG
pMsU6g2-GUS-MDC32-HindIII-F	gtaaaacgacggccagtgccaagcttGCATTGGGACTGAGATGGTGA
pMsU6g2-GUS-MDC32-KpnI-R	gtttctacaggacgtaacatggtaccAAGCCTTCGAGTTAGCTTGCAG
pMsU6g4-GUS-MDC32-HindIII-F	gtaaaacgacggccagtgccaagcttATCTCAAATGCTGATGCTACCG
pMsU6d6-GUS-MDC32-HindIII-F	gtaaaacgacggccagtgccaagcttTGACATGCTATGAAAAGTCTGC
pMsU6g4d6-GUS-MDC32-KpnI-R	gtttctacaggacgtaacatggtaccAAGCCTTCTGGTTCGCTTGCAG
pMsU6h1-GUS-MDC32-HindIII-F	gtaaaacgacggccagtgccaagcttTGTCAATTTCGACTGGCATG
pMsU6h1-GUS-MDC32-KpnI-R	gtttctacaggacgtaacatggtaccGTTAGTGAGAGTGTGCTAGCGTA
pMsU6h2-GUS-MDC32-HindIII-F	gtaaaacgacggccagtgccaagcttACGGAGATGTTTCTAATAGTTGC
pMsU6h2-GUS-MDC32-KpnI-R	gtttctacaggacgtaacatggtaccAAGCCATTCAGTGCGCTAGCG
pMsU6i1k-GUS-MDC32-HindIII-F	gtaaaacgacggccagtgccaagcttGTTTCCTGTCCAACCGTAGC
pMsU6i1k-GUS-MDC32-KpnI-R	gtttctacaggacgtaacatggtaccAATAGAAGAGAGCATTTGCGTG
pMsU6i2o-GUS-MDC32-HindIII-F	gtaaaacgacggccagtgccaagcttCCACCTGGTAATAGAAGAGCC
pMsU6i2o-GUS-MDC32-KpnI-R	gtttctacaggacgtaacatggtaccAAGCTAGGATGTTATGTAGGGT
pMsU6i3-GUS-MDC32-HindIII-F	gtaaaacgacggccagtgccaagcttTTTCTTGTTTATGACCGAGTTTC
pMsU6i3-GUS-MDC32-KpnI-R	gtttctacaggacgtaacatggtaccAAGCCCTGTTGTTCGCCTGGA
pMsU6l-GUS-MDC32-HindIII-F	gtaaaacgacggccagtgccaagcttCATGGTCAACAAAGGGTTCG

pMsU6l-GUS-MDC32-KpnI-R	gtttctacaggacgtaacatggtaccAAGCCCTGTTGTTCGTCTAGA
pMsU6m-GUS-MDC32-HindIII-F	gtaaaacgacggccagtgccaagcttCCCAAAGCCAATCCAATAAAGA
pMsU6m-GUS-MDC32-KpnI-R	gtttctacaggacgtaacatggtaccAAACCCTTTCGTTTACCTCGGC
pMsU6n-GUS-MDC32-HindIII-F	gtaaaacgacggccagtgccaagcttAATCCAATAGACACGCAATGAA
pMsU6n-GUS-MDC32-KpnI-R	gtttctacaggacgtaacatggtaccAAGCCAAACwGTCCATCTAGGC
pMDC32-HindIII-jdF	GCAAGGCGATTAAGTTGGGTA
pMDC32-KpnI-jdR	CCAGACTGAATGCCCACAGG

For vector optimization

HindIII-pMsU6d1-F	gttgtaaaacgacggccagtgccAAGCTTTCCAGCTTTAGTTTAAATAGAG
pMsU6d1-SPR-R	GTCCACTGGGTTGGTCTCCCAAGCCTTATTGTTTGCTTGATC
pMsU6d1-SPRF	GATCAAGCAAACAATAAGGCTTGGGAGACCAACCCAGTGGAC
pHSE401-R	CATGTTGACCTGCAGGCATGC
HindIII-pMsU6g1-F	gttgtaaaacgacggccagtgccAAGCTTCAGTTGAGAGCCAAAGTTCATG
pMsU6g1-SPR-R	GTCCACTGGGTTGGTCTCCCAAGCCTTCTGGTTCGCTTGTAG
SPRF	GGGAGACCAACCCAGTGGAC
HindIII-pMsU6d3-F	gttgtaaaacgacggccagtgccAAGCTTATTGAGTGGAGCGCTAAGCATTG
pMsU6d3-SPR-R	GTCCACTGGGTTGGTCTCCCACAACTTCTGGTTCGCTTGATC
HindIII-pMtU6-6-F	gttgtaaaacgacggccagtgccAAGCTTGTGTTAGCTATTTTAATTGAAG
pMtU6-6-SPR-R	GTCCACTGGGTTGGTCTCCCAAGCCTACTGGTTCGCTTGAAG
HindIII-pMtU6-5-F	gttgtaaaacgacggccagtgccAAGCTTGAGTGTCGTTTTAGTAAA
pMtU6-5-F0	GAGTGTCGTTTTAGTAAAAAAAATTATTTTAAAATGAATATCATC
pMtU6-5-SPR-R	GTCCACTGGGTTGGTCTCCCAATTTAATGGTTCGCTTGTAG
pCBC-F	GTTTTAGAGCTAGAAATAGC
pCBC-pMsU6g1-MR	CATGAACTTTGGCTCTCAACTGTATTGGTTTATCTCATCGGAAC
pCBC-pMsU6d3-MR	CAATGCTTAGCGCTCCACTCAATTATTGGTTTATCTCATCGGAAC
pCBC-pMsU6g4-MR	CAAAATCAAAGTAATAGAGAGATAATATTGGTTTATCTCATCGGAAC
pMsU6g1-F	CAGTTGAGAGCCAAAGTTCATG
pMsU6d3-F	ATTGAGTGGAGCGCTAAGCATTGAGTAAGAC
infpMsU6d1d3R1	CGACGTGAAAGCATGGTCTGTAGACAGCAAGATTT
infpMsU6d1d3F1	CAGACCATGCTTTCACGTCG
pMsU6g1-R	CAAGCCTTCTGGTTCGCTTGTAG
pMsU6d3-R	CACAACTTCTGGTTCGCTTGATC
pMsU6g4-F	TTATCTCTCTATTACTTTGATTTTG
pMsU6g4-R	CAAGCCTTCTGGTTCGCTTGCAG
pCBC-F	GTTTTAGAGCTAGAAATAGC
pCBC-R	TATTGGTTTATCTCATCGGAAC
pMsU6d1-F	GTTCCGATGAGATAAACCAATACCAGCTTTAGTTTAAATAGAG
pMsU6d1-R	CAAGCCTTATTGTTTGCTTGATC
pAtU6-F	GTTCCGATGAGATAAACCAATACGACTTGCCTTCCGCACAATAC
pAtU6-R	CAATCACTACTTCGACTCTAGC
pMtU6-6-F	GTTCCGATGAGATAAACCAATAGTGTTAGCTATTTTAATTGAAG
pMtU6-6-R	CAAGCCTACTGGTTCGCTTGAAG
pMtU6-5-F	GTTCCGATGAGATAAACCAATAGAGTGTCGTTTTAGTAAA
pMtU6-5-F0	GAGTGTCGTTTTAGTAAAAAAAATTATTTTAAAATGAATATCATC

pMtU6-5-R	CAATTTAATGGTTCGCTTGTAG
pHSE401-tRNA-F	GATCAAGCAAACAATAAGGCTTGaacaaagcaccagtggtctag
tRNA-R	tgcaccagccgggaatcgaac
tRNA-SPR-F0	gttcgattcccggctggtgcaGGAGACCAACCCAGTGGAC
pHSE401-gRNA-R	CTTGCTATTTCTAGCTCTAAAAC
pMsU6g1g4-tRNA-F	CAAGCGAACCAGAAGGCTTGaacaaagcaccagtggtctag
pMsU6d3-tRNA-F	CAAGCGAACCAGAAGTTGTGaacaaagcaccagtggtctag
tRNA-R	tgcaccagccgggaatcgaac
pCBC-F	GTTTTAGAGCTAGAAATAGC
pMsU6g1-R	CAAGCCTTCTGGTTCGCTTGTAG
pMsU6d3-R	CACAACTTCTGGTTCGCTTGATC
pMsU6g4-R	CAAGCCTTCTGGTTCGCTTGCAG
HSE401-NcoI-CmYLCV-F	atatatcctgtcaaacactgataCCATGGTGGCAGACATACTGTCCCAC
CmYLCV-KpnI-R	GGTACCaagcttagctcttacctgttttcgt
CmYLCV-KpnI-RH1-F	ggtaagagctaagcttGGTACCATGGCGAATACAAGCGGCGT
RH1-BsrGI-HSPt-R	catcttcatcttcatatTGTACATTAAAGATCTCGAAGAAATTCAAAATC
CmYLCV-KpnI-Ruby-F	ggtaagagctaagcttGGTACCATGGATCATGCGACCCTCGCCATGA
Ruby-BsrGI-HSPt-R	catcttcatcttcatatTGTACATCACTATCACTGGAGGCTTGGCTCA
BsrGI-HSPt-F	TGTACAatatgaagatgaagatgaaatatttg
HSE401-SpeI-HSPt-R	AGATTGTCGTTTCCCGCCTTCAGACTAGTcttatctttaatcatattccatag

For 2T CRISPR vector construction

PALM1-T1-A-BsF	tggtctcgATTGGGTCAGCTCAAGCTCTTGGGTTTTAGAGCTAGAAATAGC
PALM1-T1-C-BsF	tggtctcgCTTGGGTCAGCTCAAGCTCTTGGGTTTTAGAGCTAGAAATAGC
PALM1-T1-T-BsF	tggtctcgTGTGGGTCAGCTCAAGCTCTTGGGTTTTAGAGCTAGAAATAGC
PALM1-T2-d3-BsR	tggtctcgAAACCCGCGACCGTGCTCGTCTCCACAACTTCTGGTTCGCTTGATC
PALM1-T2-g1-BsR	tggtctcgAAACCCGCGACCGTGCTCGTCTCCAAGCCTTCTGGTTCGCTTGTAG
PALM1-T2-d1-BsR	tggtctcgAAACCCGCGACCGTGCTCGTCTCCAAGCCTTATTGTTTGCTTGATC
PALM1-T2-t6-BsR	tggtctcaAAACCCGCGACCGTGCTCGTCTCCAAGCCTACTGGTTCGCTTGAAG
PALM1-T2-t5-BsR	tggtctcgAAACCCGCGACCGTGCTCGTCTCCAATTTAATGGTTCGCTTGTAG
PALM1-T2-A6-BsR	tggtctcgAAACCCGCGACCGTGCTCGTCTCCAATCACTACTTCGACTCTAGC

For 3T CRISPR vector construction

TFL1C-T1-BsF	tggtctcgCTTGACACTAAGCAAGTTTACAAGTTTTAGAGCTAGAAATAGC
T0-BsR2	tggtctcaAGCCTTCTGGTTCGCTTGTAG
TFL1C-T2-BsF2	tggtctcaGGCTTGCACTAGGGCCAGGGACATCGTTTTAGAGCTAGAAATAGC
TFL1C-T3-BsR	tggtctcgAAACTCAGGTACGGATCACTAGGCACAACTTCTGGTTCGCTTGATC
TFL1A-T4-BsF	tggtctcgCTTGACAGTCATTTTCATGCTTGGTTTTAGAGCTAGAAATAGC
T0-BsR2	tggtctcaAGCCTTCTGGTTCGCTTGTAG
TFL1A-T5-BsF2	tggtctcaGGCTTGATGAGGTCCTTCTATACACGTTTTAGAGCTAGAAATAGC
TFL1A-T6-BsR	tggtctcgAAACTGATGTTCCTGGCCCTAGTCACAACTTCTGGTTCGCTTGATC
TFL1B-T7-BsF	tggtctcgCTTGCCAAGCCAAGGATTGAGATGTTTTAGAGCTAGAAATAGC
T0-BsR2	tggtctcaAGCCTTCTGGTTCGCTTGTAG
TFL1B-T8-BsF2	tggtctcaGGCTTGATATAATACCAGTGTAAAGGTTTTAGAGCTAGAAATAGC
TFL1B-T9-BsR	tggtctcgAAACTTTCCTTCTTCAGTTACCACACAACTTCTGGTTCGCTTGATC

**For 9T CRISPR vector construction**	
TFL1C-T1-BsF2	tggtctcgTGCAAACACTAAGCAAGTTTACAA
TFL1C-T1-BsF	tggtctcgTGCAAACACTAAGCAAGTTTACAAGTTTTAGAGCTAGAAATAGC
TFL1C-T2-R	tggtctcaTGGCCCTAGTGATGCACCAGCCGGGAATCG
TFL1C-T2-F	tggtctcaGCCAGGGACATCGTTTTAGAGCTAGAAATAGC
TFL1C-T3-R	tggtctcaGGATCACTAGGGTGCACCAGCCGGGAATCG
TFL1C-T3-F	tggtctcaATCCGTACCTGAGTTTTAGAGCTAGAAATAGC
TFL1A-T4-R	tggtctcaAAAATGACTGTGTGCACCAGCCGGGAATCG
TFL1A-T4-R2	tggtctcaAAAATGACTGTG
TFL1A-T4-F2	tggtctcaTTTTCATGCTTG
TFL1A-T4-F	tggtctcaTTTTCATGCTTGGTTTTAGAGCTAGAAATAGC
TFL1A-T5-R	tggtctcaAAGGACCTCATGTGCACCAGCCGGGAATCG
TFL1A-T5-F	tggtctcaCCTTCTATACACGTTTTAGAGCTAGAAATAGC
TFL1A-T6-R	tggtctcaCTGGCCCTAGTGTGCACCAGCCGGGAATCG
TFL1A-T6-F	tggtctcaCCAGGAACATCAGTTTTAGAGCTAGAAATAGC
TFL1B-T7-R	tggtctcaCCTTGGCTTGGTTGCACCAGCCGGGAATCG
TFL1B-T7-R2	tggtctcaCCTTGGCTTGGT
TFL1B-T7-F2	tggtctcaAAGGATTGAGAT
TFL1B-T7-F	tggtctcaAAGGATTGAGATGTTTTAGAGCTAGAAATAGC
TFL1B-T8-R	tggtctcaTGGTATTATATTTGCACCAGCCGGGAATCG
TFL1B-T8-F	tggtctcaACCAGTGTAAAGGTTTTAGAGCTAGAAATAGC
TFL1B-T9-BsR	tggtctcgAAACTTTCCTTCTTCAGTTACCACTGCACCAGCCGGGAATCG
TFL1B-T9-BsR2	tggtctcgAAACTTTCCTTCTTCAGTTACCAC
**For 12T CRISPR vector construction**	
TFL1C-T1-BsF2	tggtctcgTGCAAACACTAAGCAAGTTTACAA
TFL1C-T1-BsF	tggtctcgTGCAAACACTAAGCAAGTTTACAAGTTTTAGAGCTAGAAATAGC
TFL1A-T13-R	tggtctcaTCCAACAATTAGTGCACCAGCCGGGAATCG
TFL1A-T13-F	tggtctcaTGGAAGAGTGATGTTTTAGAGCTAGAAATAGC
TFL1A-T5-R	tggtctcaAAGGACCTCATGTGCACCAGCCGGGAATCG
TFL1A-T5-F	tggtctcaCCTTCTATACACGTTTTAGAGCTAGAAATAGC
BFT-T10-R	tggtctcaAATGAATGTGACTGCACCAGCCGGGAATCG
BFT-T10-R2	tggtctcaAATGAATGTGAC
BFT-T10-F2	tggtctcaCATTCTTACACT
BFT-T10-F	tggtctcaCATTCTTACACTGTTTTAGAGCTAGAAATAGC
BFT-T11-R	tggtctcaTGATCCACATTTTGCACCAGCCGGGAATCG
BFT-T11-F	tggtctcaATCACTAGGACTGTTTTAGAGCTAGAAATAGC
BFT-T12-R	tggtctcaGGATCACTAGGATGCACCAGCCGGGAATCG
BFT-T12-F	tggtctcaATCCACATTTAAGTTTTAGAGCTAGAAATAGC
TFL1B-T7-R	tggtctcaCCTTGGCTTGGTTGCACCAGCCGGGAATCG
TFL1B-T7-R2	tggtctcaCCTTGGCTTGGT
TFL1B-T7-F2	tggtctcaAAGGATTGAGAT
TFL1B-T7-F	tggtctcaAAGGATTGAGATGTTTTAGAGCTAGAAATAGC
TFL1A-T6-R	tggtctcaCTGGCCCTAGTGTGCACCAGCCGGGAATCG
TFL1A-T6-F	tggtctcaCCAGGAACATCAGTTTTAGAGCTAGAAATAGC

TFL1A-T14-R	tggtctcaTAGCATCTGTTGTGCACCAGCCGGGAATCG
TFL1A-T14-F	tggtctcaGCTACATTTGGTGTTTTAGAGCTAGAAATAGC
TFL1C-T3-R	tggtctcaGGATCACTAGGGTGCACCAGCCGGGAATCG
TFL1C-T3-R2	tggtctcaGGATCACTAGGG
TFL1C-T3-F2	tggtctcaATCCGTACCTGA
TFL1C-T3-F	tggtctcaATCCGTACCTGAGTTTTAGAGCTAGAAATAGC
TFL1A-T6-R	tggtctcaCTGGCCCTAGTGTGCACCAGCCGGGAATCG
TFL1A-T6-F	tggtctcaCCAGGAACATCAGTTTAGAGCTAGAAATAGC
TFL1B-T9-BsR	tggtctcgAAACTTTCCTTCTTCAGTTACCACTGCACCAGCCGGGAATCG
TFL1B-T9-BsR2	tggtctcgAAACTTTCCTTCTTCAGTTACCAC
**For quantitative RT-PCR analysiss**	
gRNA-R	GCACCGACTCGGTGCCAC
Hyg-rev-R	GTAGATCTCATTGATAGCTC
gRNA1-F1	GGGTCAGCTCAAGCTCTTGGGT
gRNA2-F1	GGAGACGAGCACGGTCGCGGGT
gRNA-RT	CGACTCGGTGCCACTTTTTCAAGTTG
Hyg-qF1	CTATCAGAGCTTGGTTGACGG
Hyg-qR1	CTTCTACACAGCCATCGGTC
**For genotyping analysis**	
M13F	GTAAAACGACGGCCAGT
MsU6g1-seqR	GTAGATCTCATTGATAGCTC
MsU6d3-seqR	GATTCACTCACAATTTGTGTC
MsU6g4-seqR	ATCTCACTCTCCAACCATG
pHSE401-R	CATGTTGACCTGCAGGCATGC
**For Hi-TOM sequencing**	
MsPALM1-HiTOM-F1	ggagtgagtacggtgtgcGACACAAGGAATATTATGAAC
MsPALM1-HiTOM-R1	gagttggatgctggatggGAGGAATATTTATGAATGGTG
MsTFL1A-HiTOM-F1	ggagtgagtacggtgtgcTGTCTCAAGAACCACTAATTG
MsTFL1A-HiTOM-R1	gagttggatgctggatggAAATAGAGAATAAACGTGAGAT
MsTFL1A-HiTOM-F2	ggagtgagtacggtgtgcCAACTATCATTGTGTGTGTGC
MsTFL1A-HiTOM-R2	gagttggatgctggatggCCAGTGCAAGTGTTCTCTTAG
MsTFL1A-HiTOM-F3	ggagtgagtacggtgtgcGATTGTGACAGATATTCCTGG
MsTFL1A-HiTOM-R3	gagttggatgctggatggCATAGCTCACTACTTCTTTCC
MsTFL1B-HiTOM-F	ggagtgagtacggtgtgcCAACCACAAAAATGACTGTAAC
MsTFL1B-HiTOM-R	gagttggatgctggatggCATTTCTATAATTATGTATGAGATG
MsTFL1C-HiTOM-F	ggagtgagtacggtgtgcAATGTCTGTCACTTACAACACT
MsTFL1C-HiTOM-R	gagttggatgctggatggGTTCAAGTTATATGTATAACATACC
MsBFT-HiTOM-F1	ggagtgagtacggtgtgcATGTCTAGGCCATTGGAACC
MsBFT-HiTOM-R1	gagttggatgctggatggACCCTTGGCTTGTTCATAAC
MsBFT-HiTOM-F2	ggagtgagtacggtgtgcACTTGTAGATCATGACAGACC
MsBFT-HiTOM-R2	gagttggatgctggatggCCTGGAATATCTGTAACCATC
